# Chemical Profiling, Antioxidant, and Antimicrobial Activity of Saudi Propolis Collected by Arabian Honey Bee (*Apis mellifera jemenitica*) Colonies

**DOI:** 10.3390/antiox11071413

**Published:** 2022-07-21

**Authors:** Wed Mohammed Ali ALaerjani, Khalid Ali Khan, Badria M. Al-Shehri, Hamed A. Ghramh, Ajaz Hussain, Mohammed Elimam Ahamed Mohammed, Muhammad Imran, Irfan Ahmad, Saboor Ahmad, Abdulrhman S. Al-Awadi

**Affiliations:** 1 Chemistry Department, Faculty of Science, King Khalid University, P.O. Box 9004, Abha 61413, Saudi Arabia; 442813325@kku.edu.sa (W.M.A.A.); balshehre@kku.edu.sa (B.M.A.-S.); meaahmad@kku.edu.sa (M.E.A.M.); miahmad@kku.edu.sa (M.I.); 2 Research Center for Advanced Materials Science (RCAMS), King Khalid University, P.O. Box 9004, Abha 61413, Saudi Arabia; halgramh@kku.edu.sa; 3 Unit of Bee Research and Honey Production, Faculty of Science, King Khalid University, P.O. Box 9004, Abha 61413, Saudi Arabia; 4 Biology Department, Faculty of Science, King Khalid University, P.O. Box 9004, Abha 61413, Saudi Arabia; 5 Institute of Chemical Sciences, Bahauddin Zakariya University, Multan 60800, Pakistan; drajazhussain@bzu.edu.pk; 6 Department of Clinical Laboratory Sciences, College of Applied Medical Sciences, King Khalid University, P.O. Box 61421, Abha 62529, Saudi Arabia; imahmood@kku.edu.sa; 7 Key Laboratory of Pollinating Insect Biology, Institute of Apicultural Research, Ministry of Agriculture, Chinese Academy of Agricultural Sciences, Beijing 100093, China; 2018y100172@caas.cn; 8 K.A. CARE Energy Research and Innovation Canter in Riyadh, King Saud University, Riyadh 11451, Saudi Arabia; alawadi@ksu.edu.sa

**Keywords:** honey bee products, propolis, phenolic content, biological properties, molecular docking

## Abstract

Propolis (bee glue) is a complex, phyto-based resinous material obtained from beehives. Its chemical and biological properties vary with respect to bee species, type of plants, geographical location, and climate of a particular area. This study was planned with the aim of determining the chemical composition and to investigate various properties (against oxidants and microbes) of different extracts of Saudi propolis collected from Arabian honey bee (*Apis mellifera jemenitica*) colonies headed by young queens. Chemical analysis of propolis extracts with different solvents, i.e., ethyl acetate (Eac), methanol (Met), butanol (BuT), and hexane (Hex) was done through colorimetry for the total phenolic content (TPC) and total flavonoid content (TFC) evaluation. For separation and extensive characterization of the Met extract, chromatography and ^1^H NMR were deployed. Six different microorganisms were selected to analyze the Saudi-propolis-based extract’s antimicrobial nature by measuring zones of inhibition (ZOI) and minimum inhibitory concentration (MIC). Molecular docking was done by utilizing AutodDock, and sketching of ligands was performed through Marvin Chem Sketch (MCS), and the resultant data after 2D and 3D clean were stored in .mol format. The highest TFC (96.65 mg quercetin equivalents (QE)/g of propolis) and TPC (325 mg gallic acid equivalents (GAE)/g of propolis) were noted for Met. Six familiar compounds were isolated, and recognition was done with NMR. Met extract showed the greatest 1,1-diphenyl-2-picrylhydrazyl radical (DPPH) free radical scavenging activity and Ferric Reducing Antioxidant Power (FRAP). Met showed max microbial activity against *Staphylococcus aureus* (ZOI = 18.67 mm, MIC = 0.625 mg/mL), whereas the minimum was observed in Hex against *E. coli* (ZOI = 6.33 mm, MIC = 2.50 mg/mL). Furthermore, the molecular docking process established the biological activity of separated compounds against HCK (Hematopoietic cell kinase) and Gyrase B of *S. aureus*. Moreover, the stability of protein–ligand complexes was further established through molecular dynamic simulation studies, which showed that the receptor–ligand complexes were quite stable. Results of this research will pave the way for further consolidated analysis of propolis obtained from Arabian honey bees (*A. m. jemenitica*).

## 1. Introduction

Beehive resin, known as propolis or bee glue, is a non-toxic and resinous substance. Bees bring it back to their hive after foraging on a variety of plants. The color of propolis may vary from creamy, yellow, green, light to dark brown, or even sometimes so dark that it resembles asphalt [1,2]. Propolis possesses a complex composition, and its chief components are resin (50%), beeswax (30%), aromatic compounds, essential oils (10%), pollen (5%), and the remaining 5% are diverse organic compounds such as polyphenols, flavonoids, amino acids, vitamins, and micronutrients [3]. Wax is a glandular bee secretion, and bees collect resin for propolis production, mainly from plant buds and exudates. Marcucci et al. [4] and Bankova et al. [5] have reviewed the types of substances in propolis, and they confirmed the presence of more than 300 known constituents in propolis.

Honey bees use this safeguard product for filling in ceiling holes, cracks for reconstruction purposes, covering cadavers of attackers, and strangulating the inner layers of beehives. This buffers the inner temperature of the hive at around 35 °C and aids in haltering the entry of intruders. Comb hexagonal cells in the walls of beehives possess a mixture of bee wax and propolis. It has been established that, in addition to hardening hive cell walls, this valuable product contributes to achieving an internal sterile environment [2,6,7]. Propolis and its other extracts are widely used owing to their medicinal and therapeutic attributes. They are employed against various inflammations [8], as oxidants [9,10], and for their antimicrobial benefits [10,11]; their immunomodulatory characteristics are also being deciphered in the medical sector [5]. The antiseptic efficiency of propolis was established ages ago. Aristotle used it to treat abscesses and other wounds. Its antipyretic properties were well-known to primitive Arabs, Incas, Egyptians, Romans, Chinese, and Greeks, who used it to treat various wounds [6,12,13].

In the modern era, propolis gained appreciation as an effective means of treating health problems. In 1985, propolis was first used in pharmacology due to its promising treatment attributes [2]. Research on propolis had also proposed its botanical origin and vast variations present in its chemical composition when samples from different or even the same location were compared. South American or European-based propolis share characteristics such as having anti-viral, antimicrobial, wound-palliating, immune-stimulating, anti-inflammation, and hypnotic attributes. Regardless of their similar characteristics, the plants that provide resin for the synthesis of propolis on both continents (South America and Europe) have different chemical compositions [14]. Flavonoids have been identified as the major pharmacologically active constituent of European propolis, with galangin being the most common and abundantly expressed among flavonoids [15,16]. According to research, the distinct chemical nature of this European propolis is due to European bees collecting resin for propolis production from the buds of Populus nigra, Salicaceae [17]. Oxidative stress occurs during various biochemical disorders that lead to the synthesis of different reactive oxygen species (ROS) and that may ultimately lead to death. Antioxidants are described as effective scavengers of these ROS by minimizing cell damage due to oxidative stress [18]. Previously, different chemical structures of propolis have been examined for their therapeutic application, and many of them have been characterized as potential antioxidant therapeutic substances. However, there has not been a lot of in-depth research into the molecular docking of Saudi-based propolis.

Most of the studies in the literature have investigated the antimicrobial activity of the propolis produced by *A. mellifera*. However, little is known about the biological effects of the propolis produced by Arabian honey bee. *A. m. jemenitica* is the smallest bee race of *A. mellifera* and native to Saudi Arabia. It can forage on plenty of bee flora which mostly remains unattended by other honey bee races. Most of the studies in the literature have investigated the chemical composition, biological activities, and antimicrobial activity of the propolis samples collected by other honey bee races rather than Arabian honey bee and from various other geographic areas. However, very little is known about the chemical composition, biological effects, and antimicrobial properties of the propolis collected by the Arabian honey bee from the Asir region of Saudi Arabia. Although it is obvious that the propolis is a plant-originated bee product, its chemical composition, physical properties, and biological activities vary according to available local flora, harvesting season, and type of bee species [19,20]. Due to the adeptness in the local environment, foraging behavior on local flora, and morphological characteristics of Arabian honey bee, we hypothesize that the propolis produced by this race has distinctive composition and biological activity. Thus, the aim of this work was to investigate the chemical composition, biological activities, and antimicrobial potential of propolis samples collected from the Asir region of Saudi Arabia. The findings of this work might be important for future uses of propolis in clinics to combat antibiotic resistant pathogens.

## 2. Materials and Methods

### 2.1. General

Analytical grade hexane, dichloromethane (DCM) and absolute alcohol were bought from a Darmstadt, Germany-based company (Merck) for fractionation and purification purposes, whereas reagent 2,2-diphenyl-1-picryl-hydrazyl was taken from another German company, Sigma-Aldrich. Methanol HPLC grade was taken from Pronolab (Lisbon, Portugal). The other chemicals were obtained from an American-based company (Sigma-Aldrich) for antimicrobial, antioxidant, cytotoxic activities. A mixture of deuterated solvents and internal standard tetramethyl silane was provided by American-based Cambridge Isotope Laboratories. For silica gels having 230–400 mesh, E. Merck was used to perform column chromatography, and for detection of compounds, UV radiations in the range of 254 nm to 366 nm were employed together with the spraying of ammonium cerium (IV) sulphate-hydrate in ten percent H_2_SO_4_ (heating) or ninhydrin. Various instruments were taken from a Tokyo-based, Japanese company (Shimadzu Corporation), including Shimadzu version UV-240 (spectrophotometer for Spectra of UV) and Shimadzu IR-460 (for ascertaining spectra of IR) by KBr pellet sample formation method). To carry out NMR (Nuclear magnetic resonance), a Spectrophotometer (Bruker AVANCE III, 400 MHz) was deployed by using the internal standard as TMS. A double focusing mass spectrometer (Varian-MAT 112S) was used to note mass spectra. Ions were estimated in m/z (%).

### 2.2. Saudi Propolis Sample Collection and Extraction

Propolis was taken from 25 beehives of local honey bee (*Apis mellifera jemenitica*) colonies headed by healthy one-year-old young queens, which were located in Al-Souda Abha, Saudi Arabia, using the procedure given by [21]. A stainless-steel spatula was used to harvest propolis from all investigated beehives, kept in Falcon tubes (50 mL), and then stored in the freezer for further analysis. Four solvents, named *n*-hexane, *n*-butanol, ethyl acetate, and methanol were employed for extraction purposes. A volumetric flask (250 mL) was taken for each of the solvents, and 20 g of propolis was added. The flask was then filled with the corresponding solvent (200 mL, 99% *v*/*v*). This blend was shaken at high speed of 180 rpm for 24 h at room temperature. For filtering of the resultant suspension, Whatman filter paper (No. 1, Sigma-Aldrich, St. Louis, MO, USA) was used. Extraction of further residues was done according to the procedure described by [1]. Pooling of filtrates from both extracts was done and kept at minus 20 °C for a day for precipitation of resin and wax. The obtained suspension was subjected to centrifugation, and then vaporization of clear supernatant was carried out in a rotary evaporator maintained at 40 °C. For further analysis, one gram of each propolis extract (dry form) was mixed in 100 mL of methanol and then frozen (Sigma-Aldrich, St. Louis, MO, USA) (stock solution) for use in further analysis. Propolis was present in the stock solution at a concentration of 10 mg per milliliter of methanol.

#### Method to Extract, Fractionate, and Isolate Compounds **1**–**6**

Chemicals such as *n*-butanol, methanol, ethyl acetate, and *n*-hexane were used to extract air-dried samples in powder form, and this was done for seven days, three times each. The resultant extract was concentrated in vacuo by using rotavapor. Centered on inceptive biological screening, VLC (Vacuum column chromatography) was conducted by using normal phase silica gel on the methanol ethanol-soluble extract, and then elution was done with a gradient mixture of *n*-hexane-DCM-methanol for fractionation (M1-M6). M1 fraction, which was eluted with H-DCM (*n*-hexane-dichloromethane), was subjected to chromatography by deploying silica gel CC to obtain compounds **1** and **2**. The fraction M3 fraction was isolated by CC utilizing H-DCM (*n*-hexane-dichloromethane) to get compounds **3** and **4**. In comparison, fraction F7 was further utilized to get 3 major fractions (E1-E3) by subjecting it to chromatography on silica gen utilizing an H-DCM gradient system. The compounds **5** and **6** were achieved through E2 (Subfraction) using CC and the identical mobile system. Based on experimental data, these compounds are named as 4-methyl salicylic acid 1 (CLR: light yellow crystal, MP:179 °C, MF: C8 H8 O3), Cinnamic acid 2 (CLR: White crystal, MP:135 °C MF: C9H8O2), Chrysin 3 (CLR: yellow crystal, MP:283 °C, MF: C15H10O4), Gallic acid 4 (CLR: yellowish crystal, MP:179 °C, MF: C7H6O5), Apigenin 5 (CLR: yellow amorphous, MP: 345–348 °C, MF: C15H10O5), and Myricetin 6 (Color (CLR): pale yellow crystal, Melting Point (MP): 300 °C, Molecular Formula (MF): C15H10O8) (Supplementary Table S1).

### 2.3. Determination of Total Phenol Content (TPC)

A colorimetric assay, according to standard procedure as delineated by Kumazawa et al. [22] and Singleton et al. [23] with slight modifications, was utilized to determine the total quantity of phenols/flavonoids in the methanolic extract of various propolis. The reaction mixture consisted of a methanolic extract solution (0.5 mL), Folin-Ciocalteau (FC) reagent (0.5 mL), and 10 percent Na_2_CO_3_ (0.5 mL) and was placed in darkness at room temperature for 60 min. Afterwards, its absorbance measurement was done at 700 nm. Concentrated methanolic extract or different fractions samples (20 mg/mL) were tested and estimation was carried out with the gallic acid standard solutions calibration curve. TPC was manifested as mg of GAEs (gallic acid equivalents) per gram of extract.

### 2.4. Determination of Total Flavonoid Content (TFC)

Total flavonoid content was determined by following the procedure adopted by Mohammed et al. [24] with some modifications. Flavonoids react with Aluminum Chloride to form a colored complex compound which absorbs light at the wavelength of 415 nm. A volume of 2 mL of 2% (*W*/*V*) Aluminum chloride was added to 2 mL of propolis methanolic solution (20%, *W*/*V*), and the absorbance was read at 415 nm after 30 min of incubation. The absorbance of the propolis samples was read against a blank solution, which is composed of 2 mL methanol and 2 mL of Aluminum chloride. A quercetin standard curve was used for the calculation of the flavonoid concentration in the propolis samples. A stock solution of quercetin (0.05 mg/mL) was serially diluted up to the concentration of 0.003 mg/mL. The equation of the straight line of the standard curve was applied to calculate the concentration of the flavonoid concentration in the studied samples.

### 2.5. Procedure for 2,2-Diphenyl-1-Picrylhydrazyl (DPPH) Assay

The DPPH method as described by Anandjiwala et al. [25] with bit modifications were utilized to estimate the free radical scavenging activity (FRSA) of four Saudi-based propolis extracts (Hex, Eac, BuT, and Met). These propolis samples exhibited a decrease in absorbance value (λ = 517 nm) of colored solution of DPPH methanol depicting the FRSA activity of study samples [26,27,28]. The stock solution was prepared by dissolving 4 milligrams of DPPH that was weighted on the watch glass in 100 milliliters of 99 percent methanol. In a similar way, the stock solution for each extract of propolis was composed by deliquescing 1 gram of propolis extract in 100 mL of 99 percent methanol. Then, this stock solution of propolis extract was used to make their respective serial dilutions of 5, 10, 20, 40, 60, 80, 100, 150, 200, and 250 micrograms per milliliter. Standard ascorbic acid (SAA) was used as a reference, and it was dissolved in 99 percent methanol to provide a stock solution containing identical amounts of Met, BuT, Hex, and Eac. One milliliter of reference and every serial of propolis extract (that has been diluted) was added in discrete test tubes of 10 mL. Afterwards, 4 milliliters of DPPH methanol solution were put in test tubes, stirred well, and then methanol was added to make the final volume of tubes up to10 mL. Then, for about 30 min, this reaction mixture was placed in darkness. For UV/Vis, a spectrophotometer was deployed to record absorbance (517 nm). HPLC-grade, at 99 percent methanol, was employed as a blank.

Control samples were constituted to contain a similar volume without propolis extract and ascorbic acid (standard). At the same time, suitable blanks and standards were run. IC_50_ was estimated from percent inhibition, and DPPH free radical scavenging activity (percent) was ascertained by the equation given below:

Inhibition in percentage = [(Ab control − Ab sample)/Ab control] × 100

Ab control denotes DPPH’s absorbance value in the above equation, and the Ab sample exhibits DPPH’s absorbance accompanied by extracts of varying concentrations.

### 2.6. Ferric Reducing Antioxidant Power (FRAP)

A Ferric Reducing Antioxidant Power (FRAP) assay was used to access the reduction power of the propolis extracts. The basic principle of the assay involves reduction of Fe + 3 to Fe + 2. The iron ions are bound to 2,4,6 tripyridyl-S-triazine (TPTZ). The FRAP reagent was composed of 0.3 M acetate buffer (10 mL), 0.01 M TPTZ in 0.04 M HCl (1 mL) and 0.02 M FeCl_3_. 6H_2_O (1 mL). A volume of 1.5 mL of the FRAP reagent was added to 200 µL of propolis extract solution (1 g in 7 mL methanol) and incubated at 37 °C for 4 min. The absorbance was read at 593 nm after calibrating the spectrophotometer using 200 µL of distilled water in state of Propolis sample. The standard curve was created by reacting ferrous sulfate (151.5–9.5 mg/mL) with the FRAP reagent [29].

### 2.7. Antimicrobial Activity Assay

#### 2.7.1. Well-Diffusion Method

To decipher the possible antimicrobial prospects of Hex, BuT, Eac, and Met, different strains of bacteria (Gram-negative, Gram-positive) and fungi were selected. Gram-negative bacteria include *Escherichia coli*, *Shigella flexneri*, *Proteus mirabilis* denoted by ATCC 25922, ATCC 12022, and ATCC 29906, respectively. Gram-positive bacteria include *Staphylococcus aureus,* and *S. epidermidis* symbolized by ATCC 29,213 and ATCC 12,228 respectively, whereas a single fungus (*Candida albicans* denoted by ATCC 7596) was included in the current study. Agar slants with proper nutrients were maintained at 4 °C to retain strains of microbes under analyzation. These microbial strains were activated by inoculation of every microbial strain (10 mL) broth and kept in an incubator at 37 °C for a night before the assay. Broth solution alongside nutrient agar was constituted as per standard prescriptions. A laminar flow hood was used in preparation of agar plates by pouring autoclaved nutrient agar that has been autoclaved into plates. Inoculation of the surface of agar plates was carried out by spreading (with sterile cotton) each microbial suspension (50 µL). A sterile cork borer was employed to punch 5 holes of 6 mm in diameter in each of the agar plates. In total, 30 µL of extract of propolis (at a quantity of 10 mg/mL, *W*/*V*) of each type was added to the wells of plates containing agar. Penicillin-Streptomycin = 20 units: 20 microgram solution was utilized as the positive control. All incubation of petri plates at 33 °C for 24 h was carried out. The procedure given by Ghanem [30] was used to determine the antimicrobial inhibition by computing the diameter of the inhibition zone in a millimeter set up around each analyzed propolis extract. Experiments were conducted in triplicate fashion, and the average of 3 values was computed and utilized in statistical procedures. Various strains of microbes employed in the current research were obtained from the Microbiological Laboratory located in the Department of Biology, King Khalid University, Abha, KSA.

#### 2.7.2. Determination of Minimum Inhibitory Concentration (MIC)

MIC of various propolis extracts were ascertained [31]. Amounts of the extracts utilized for MICs on the microbial isolates were treated with 2-fold dilution of extracts ranging from 10 mg/mL to 0.078 mg/mL. In order to find out the MIC, the microbial strains were developed to the logarithmic phase (0.4–0.6 at O.D.610) and subjected to more dilution in an MH broth to a hypothetical level of O.D.610 of 0.01. Subsequently, a 180 μL culture containing all microbial strains was put inside the polystyrene sterile flat-bottom wells of 96-well plates. The wells were further subjected with 20 μL of 2-fold dilution of various propolis extracts. The wells loaded with 20 μL of DMSO were deliberated as control. Further incubation of the plates was done aerobically for almost 24 h at 37 °C [32]. Then, 20 μL of alamar blue dye (Thermo Fisher, Waltham, MA, USA) was put into each well after subjecting it to an incubation period of 24 h, and after each hour, the development of pink color was checked. The lowest concentration of the extracts in a particular well where the color was unchanged was considered as MIC.

### 2.8. Method of Molecular Docking

In silico explorations were carried out on AutodDock ver1.5.6 [33]. The compounds **1**–**6** (ligands), as shown in Table S1, were sketched by MCS (Marvin chem sketch) and the files for ligands with .mol extensions after cleaning the ligand in 2D and 3D. UCSF Chimera was deployed for preparation of ligand molecules using the dock prep tab of chimera and the files were saved in .mol2 format. The ligand files with a .mol2 extension were used for fixing the number of torsions and generation of .pdbqt files from .mol2 files through autodock tools (ADT version 1.5.6).

Hematopoietic cell kinase crystal structure bearing pdb ID 2HCK and *S. aureus* Gyrase B (pdb ID 5D6P) were redeemed from the protein database (http://www.rcsb.org: accessed on 8 March 2022) in .pdb format. Similarly, the CS (crystal structure) of GyraseB was also obtained from the protein database (pdb 5D6P). Molecular docking was performed after removing water and ligand molecules that were co-crystalized. UCSFC Chimera was deployed for adding H atoms and gasteiger charges for the 3D conformation belonging to the macromolecules of 2HCK and 5D6P, and resultant receptor files were also saved in pdb format. Autodock was utilized to generate. pdbqt and grid boxes. To cover the binding site of 2HCK, a grid box was designed with the dimensions of 40 × 40 × 40 xyz points and a spacing of 0.357 °A. Likewise, for 5D6P, the grid box was prepared with the dimensions of 34 × 34 × 34 xyz points and a spacing of value 0.35A. Redocking of molecules (that are co-crystallized) was successfully achieved to predict the binding pose of native ligands on the active site of macromolecules which was further used to set the dimensions of a suitable grid box. Later on, docking of ligands **1**–**6** was accomplished using Vina through command prompt.

### 2.9. Statistical Analysis

Analysis of variance (ANOVA) was used, followed by a mean *t*-test to analyze data collected from performed biological experiments using statistical software and a statistical analysis system (SAS). The Tukey (Honest Significant Difference) test was performed for multiple comparisons based on means. The level of significance was 5% (0.05), so values less than 0.05 were regarded as significant values. Likewise, numerical data was also inferred from propolis fractions (3 replications) for their respective antimicrobial behavior measured in average diameter of the inhibition zone (ZOI). Standard deviation was also computed. For antimicrobial data, ANOVA was made using Statistix8.1 software. The above-mentioned Tukey test was performed for pairwise mean comparison. This means that *p* ≤ 0.05 was measured as statistically significant.

Probability values smaller than 0.05 were accepted as statistically significant. Similarly, propolis fractions’ antimicrobial activity (expressed in mean diameter of inhibition zone) was measured from three replicates along with the standard deviation (SD). ANOVA was performed through Statistix 8 Ver. 8.1 (Analytical Software, Tallahassee, FL, USA). A pairwise assessment to compare all the means was achieved with abovementioned Tukey test. Means that depicted differences at *p* ≤ 0.05 were considered statistically significant.

## 3. Results

**Figure 1:**
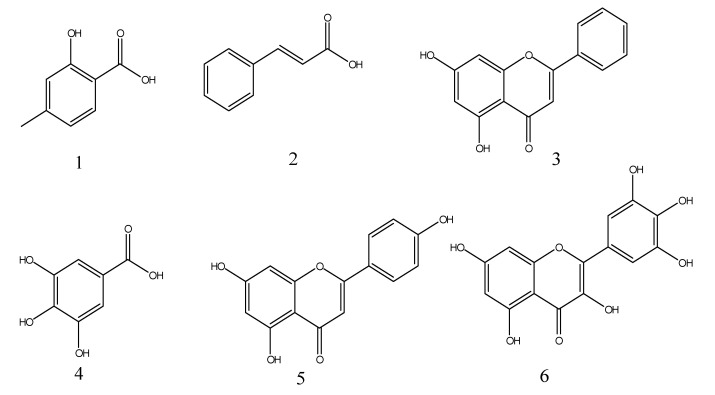
Structural composition of isolated compounds 4-methyl salicylic acid. (**1**) Cinnamic acid (**2**) Chrysin, (**3**) Gallic acid, (**4**) Apigenin, (**5**) Myricetin, and (**6**) from propolis methanol fraction.

Based upon investigations of the biological inhibition by Met obtained from Saudi-based propolis, the fraction was further analyzed by the procedure of column chromatography by deploying an organic mobile phase that led to the separation of six familiar compounds (Figure 1).

Structural features of isolated compounds were elucidated using techniques such as NMR, IR, UV, and a comparison was made between spectroscopic and physical data. These compounds were identified as 4-methyl salicylic acid **1**, Cinnamic acid **2**, Chrysin **3**, Gallic acid **4**, Apigenin **5**, and Myricetin **6** as depicted in Supplementary Table S1 [34,35,36,37].

### 3.1. Total Phenolic Contents (TPC)

Table 1 denotes the TPC for Hex, Met, Eac, and BuT. In the current study, TPC varied among different Saudi-based propolis extracts. Met showed the highest quantity (325 mg GAE/g of extract) of these compounds whereas BuT delineated the lowest concentration (271 GAE/g of extract) of this chemical substance.

### 3.2. Total Flavonoid Contents (TFC)

Total flavonoid contents, as shown in Table 1, varied in the range of 34.04–96.65 mg QE/g of propolis extracts under analysis. Hex depicted the lowest value for TFC, whereas the highest value of TFC was noted for Met.

### 3.3. Free Radical Scavenging Activity

**Figure 2:**
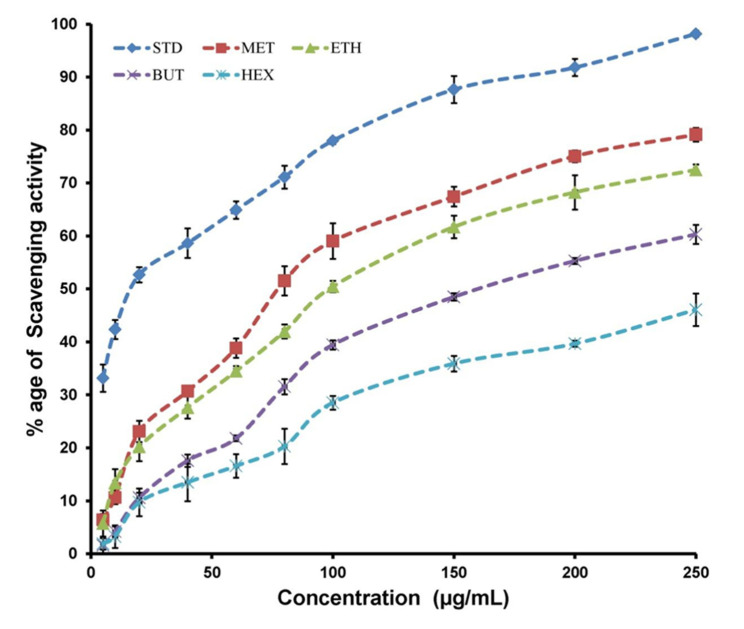
Consequences of quantity (µg/mL) on antioxidant behavior of different Saudi-propolis based extracts (HEX = hexane, ETH = ethyl acetate, BUT = butanol, MET = methanol) and CTL = Control.

Four extracts of propolis, i.e., Hex, Eac, BuT, Met, were evaluated for their antioxidant nature. For this purpose, various concentrations of four extracts ranging from 5 to 250 μg/mL were taken, as has been depicted in Figure 2 and Table 2. Significant antioxidant nature was observed for Met at a probability level of 5%. This also demonstrated higher antioxidant activity at various concentrations (60, 80, 100, 150, 200, and 250 μg/mL) compared to the other three extracts. Statistically alike antioxidant behavior was noted for Met and Eac when employed in concentrations of 20 and 40 μg/mL.

The lowest antioxidant activity was observed for Hex as compared to the other three extracts. The antioxidant behavior of BuT extracts was intermediate as it performed better against oxidants as compared to Hex, whereas Met and Eac outperformed it in this respective regard. The antioxidant activity was noted by utilizing the DPPH method, and for standardization purposes, ascorbic acid was utilized. A dose-dependent uprise in % antioxidant activity was seen for all extracts under analysis. Ascorbic acid, Met, BuT, and Eac displayed % inhibition in the order of 52.67, 51.51, 55.27, and 50.45 when 20, 80, 200, and 100 μg/mL of concentration was employed, respectively. The ascorbic acid depicted IC_50_ value was 17.72 μg/mL, and in extracts of propolis, a minimum IC_50_ was noted for Met (111.36 μg/mL), whereas the highest value for IC_50_ was observed for Hex (249.01 μg/mL). Significant DPPH radical scavenging attributes for propolis extracts under analysis are described in Table 2 and Figure 2.

### 3.4. Antimicrobial Activity

**Figure 3:**
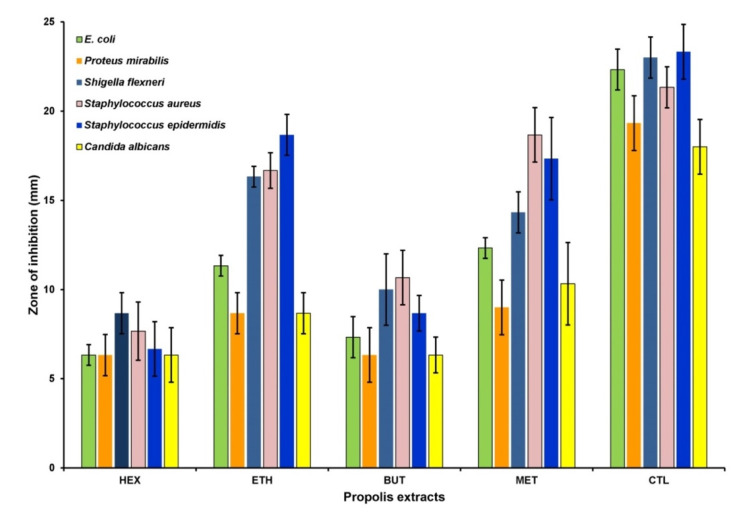
Antimicrobial capability of various propolis extracts (HEX = hexane, ETH = ethyl acetate, BUT = butanol, MET = methanol, and CTL = Control). ZOI is displayed as the average of 3 values. Error bars depict standard deviation (SD) of means.

**Figure 4:**
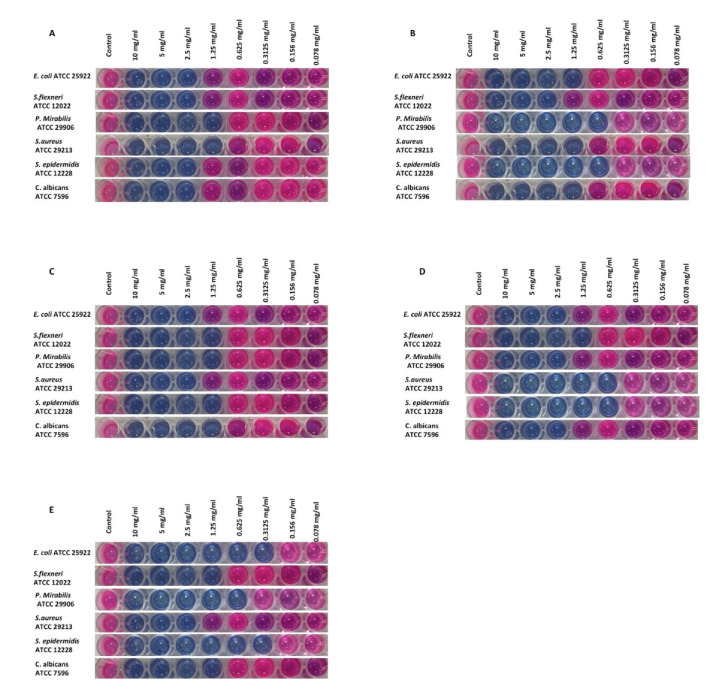
Effect of various propolis extracts with Hex = hexane solvent. (**A**) Eac = ethyl acetate solvent, (**B**) BuT = butano solvent, (**C**) Met = methanol solvent, (**D**) CTL = Dimethyl sulfoxide (DMSO) as control, and (**E**) against microbial strains.

Figure 3 depicts the findings regarding antimicrobial attributes of different Saudi propolis-based extracts. Hex and BuT are statistically alike in their characteristics against tested microorganisms and possess lower antimicrobial activity as compared to Eac and Met. The Met extract displayed the strongest antimicrobial nature against *P. mirabilis*, *S. aureus*, and *C. albicans*, whereas this activity was comparatively lower against *S. epidermis*. Eac and Met are equally effective against tested strains such as *E. Coli*, *Shigella flexneri*, and *S. epidermis*, and the activity of Eac is lower than Met but resembles other two extracts (BuT & Hex) in reaction to strains *P. mirabilis* and *C. albicans*. This research depicted the exceptional AMA of tested extracts of propolis. Met and Eac possess a relatively high antimicrobial nature as compared to Hex and BuT. In order to investigate the MIC, the selected microbial isolates were exposed with aforementioned volume of various propolis extracts, was after which an incubation period of 24 h ensued. The persistence of alamar blue was considered as the lowest concentration of the extracts and it was considered as the MIC (Figure 4). It is apparent from Figure 4 that all microbial isolates were notably susceptible to all the propolis extracts. Inhibition of bacterial growth was achieved at MIC ranging from 0.3125 to 2.5 mg/mL. Support for these above results were achieved with a subsequent higher inhibition zone (Table 3).

### 3.5. Molecular Docking

#### Receptor–Ligand Interactions

All isolated **1**–**6** flavonoids, depending on their potential activity, were subjected to docking with 2HCK (Haematopoietic cell kinase) and 5D6P (bacterial Gyrase B) to evaluate their binding interactions and effectiveness for antioxidant activity and antimicrobial activity, respectively. To visualize the interaction of ligands with macromolecules, .log (result) files were converted to .pdb file through pymol and were loaded to the Biovia Discovery Studio Visualizer (BDSV) to explore the binding design. Molecular docking was done with the objective to enhance our understanding of the receptor’s and ligand’s molecular interaction. The functions of proteins excessively depend upon a set of significant amino acid residues (Active sites) involved in interactions with the ligands.

**Figure 5:**
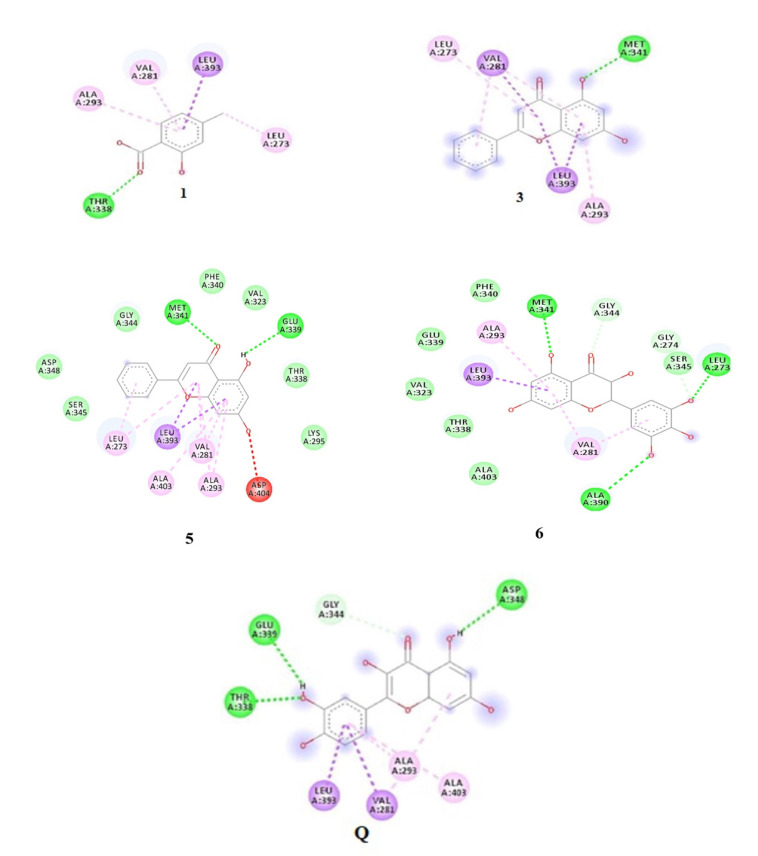
Molecular docking findings of 2D interlinkage of compounds **1**, **3**, **5**, and **6**, with the native ligand showing as Q. Green dashes exhibit conventional H-bonds.

The residues of Kinase 2CHK, including Val281, Met341, Leu393, Thr338, Gly344, Ala403, Leu273, Asp404, and Ala293, are regarded as residues of the active site and are associated with the ligand–receptor interaction. The estimated docking score for 1–6 was in the order of −6.2, −6.0, −8.5, −6.0, −8.5, and −9.2 Kcal/mol, respectively. The ligand interactions of compounds **1**–**6** have been presented in Figure 5.

**Figure 6:**
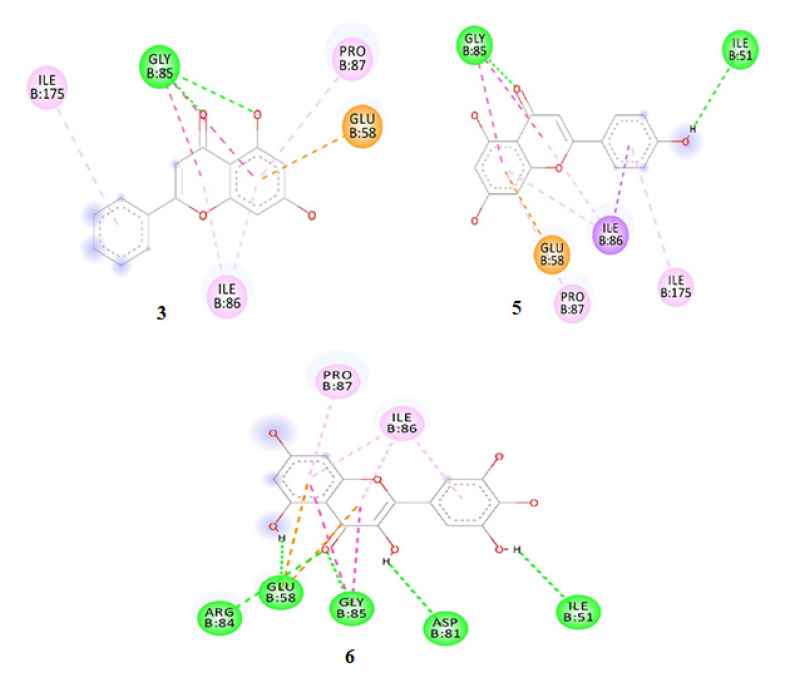
Receptor–ligand interplay of compounds such as **3**, **5**, and **6** with receptor 5D6P (Gyrase B of *S. aureus*).

The binding energies of all six compounds with 5D6P were estimated in the following order: −5.5, −5.9, −8.3, −6.0, −7.6, and −8.3 kcal/mol for compounds **1**–**6**, respectively. The ligand interactions of the isolated compounds with the protein 5D6P have been presented in Figure 6.

### 3.6. Molecular Dynamic Simulations

The molecular docking analyses showed the details of the complex binding modes (protein-inhibitor), but the smallest discrepancy can be assessed by simulating molecular dynamics. The docked complexes of most active compounds with 5D6P and 2HCK obtained from Autodock results were selected for performing the molecular dynamic simulation using the Desmond module of the Schrodinger suite [38]. Active complexes were simulated for 100 ns of the timescale using Isothermal-Isobaric ensemble (NPT ensemble) and employing a Nose-Hoover thermostat to maintain temperature at 300 K, and the pressure employed for simulation studies was 1.01325 bars, as controlled by the Martyna-Tobias-Klein barostat [39]. The desired protein–ligand complexes were saturated and partial charges were determined. Energy minimization was performed using the OPLS_2005 force field. The molecular system was solvated with water molecules with an approximate 10 Å buffering distance between the protein and the edges of the orthorhombic box [40]. The system was neutralized by adding the appropriate counter ions. For the short-range columbic interactions with a 9.0 Å radius cut-off and long-range electrostatic interactions, the smooth particle mesh Ewald method were used. In this context, one thousand steps of steepest descent energy minimization followed by conjugate gradient algorithms were utilized. RESPA (Reference system propagator algorithms) integration was used for bonded and non-bonded interaction. Three dimensional interactions were generated using Maestro graphical interface.

#### 3.6.1. MD Simulation of Compounds **5** and **6** with 5D6P

**Figure 7:**
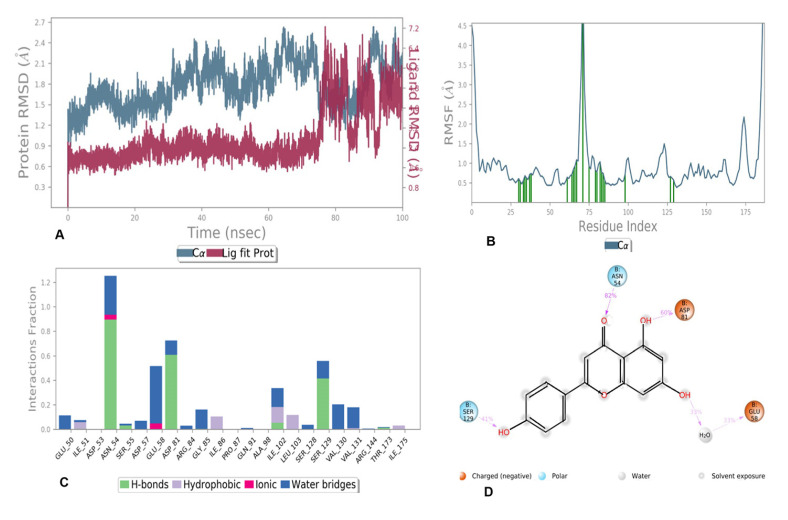
MD simulation results for compound **5** with receptor 5D6P: (**A**) Time-dependent protein-ligand root mean square deviation (RMSD) plots, (**B**) Root mean square fluctuation (RMSF) plots, (**C**) Simulation interactions with bar diagram indicating the fold of interaction, fraction, and contacts, and (**D**) 2D binding interactions.

**Figure 8:**
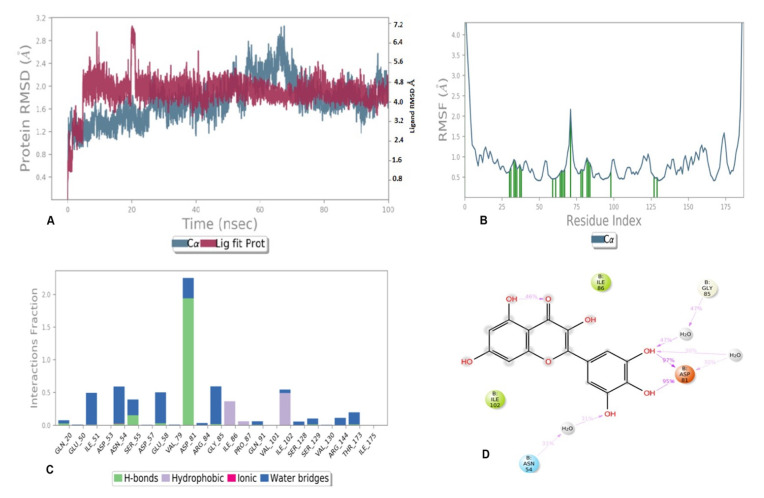
MD simulation results of compound **6** with receptor 5D6P: (**A**) Time-dependent protein-ligand root mean square deviation (RMSD) plots, (**B**) Root mean square fluctuation (RMSF) plots, (**C**) Simulation interactions with bar diagram indicating the fold of interaction, fraction, and contacts, and (**D**) 2D binding interactions.

In the case of compound 5 complexed with 5D6P, the total system consisted of 7315 water molecules and 25,020 atoms, whereas in the case of compound 6, the total system consisted of 7315 water molecules and 25,007 atoms. The fluctuation and stability of the receptor–ligand complexes during simulations in each case was analyzed and a resulting trajectory was made with backbone root mean square deviation. The metrics of root-mean-square deviation (RMSD) for Cα, backbone, and the heavy atoms of protein and the have been presented in Figure 7A for compound **5** with 5D6P. The flexibility of residues on ligand binding was analyzed using metrics of root mean square fluctuations (RMSFs) in Figure 7B. Subsequently, the ligand–receptor interaction showed the favorable contacts of the ligand with Glu50, Ile51, Asp53, Asn54, Ser55, asp57, Glu58, Asp81, Ile102, Ser129, Val130, and Val131 as depicted in Figure 7C. Lastly, the 2D trajectory of compound 5 is shown in Figure 7D. The timeline representation of protein–ligand contacts is outlined in Figure 8.

Similarly, the metrics of root-mean-square deviation (RMSD) for Cα, backbone, and the ligand have also been presented in Figure 8A for compound 6 with receptor 5D6P. The flexibility of residues on ligand binding was analyzed using metrics of root mean square fluctuations (RMSFs) in Figure 8B. The favorable contacts of six with residues of 5D6P have been presented in Figure 8C. The ligand–receptor interaction showed the favorable contacts of the ligand with Gln20, Glu50, Ile51, Asp53, Asn54, Ser55, asp57, Glu58, Val79, Asp81, Gly85, Val101 Ile102, Ser129, Val130, and Ile175, as depicted in Figure 8C, whereas the 2D trajectory of the same has been presented in Figure 8D.

The 2D trajectory diagram depicts that hydroxyl groups at C-3 and C-4 of the phenyl ring donated two conventional hydrogen bonds to Asp81 (most active residue) with 97% and 95% of simulation time whereas another hydrogen bond was accepted by the hydroxyl group at C-5 of the phenyl ring through water contact from Asn54. Ile86 and ile102 stabilized the complex through hydrophobic contact, as seen in Figure 8D.

#### 3.6.2. MD Simulation of Compounds **5** and **6** with 2HCK

**Figure 9:**
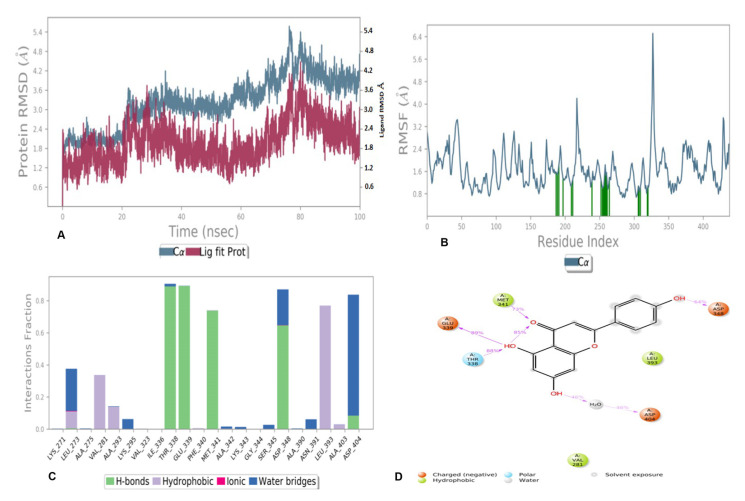
MD simulation results of compound 5 with receptor 2HCK: (**A**) Time-dependent protein-ligand root mean square deviation (RMSD) plots, (**B**) Root mean square fluctuation (RMSF) plots, (**C**) Simulation interactions diagram with bar diagram indicating the fold of interaction, fraction, and contacts, and (**D**) 2D binding interactions.

The compounds 5 and 6 were also simulated with 2HCk to evaluate the structural constancy and binding site adaptations to the docked ligand. MD simulation was performed for both the compounds for 100 ns. The stability and fluctuations of the protein and ligand alone and in complex during the simulation were investigated and the resulting trajectory for the complex was made with the backbone root mean square deviations (RMSDs), which is the average displacement of atoms from a particular frame to a reference frame. The metrics of root-mean-square deviation (RMSD) for Cα, backbone, and the heavy atoms of protein 2HCK and compound 5 have been presented in Figure 9A. The flexibility of residues on ligand binding was analyzed using metrics of root mean square fluctuations (RMSFs) in Figure 9B. Root mean square fluctuation (RMSF) was studied to recognize the critical residues involved in the major interactions with a ligand. The protein residues forming essential hydrogen bond with bound compound are represented by the green bar during the 100 ns simulation. Hydrogen bonding interactions were observed with Thr338, Glu339, Met341, Asp348, and Asp404 along with other non-bonded interactions with Leu273, Val281, Ala293, Lys295, and Leu393, as depicted in Figure 9C). Lastly, the 2D trajectory of compound 5 is shown in Figure 9D.

**Figure 10:**
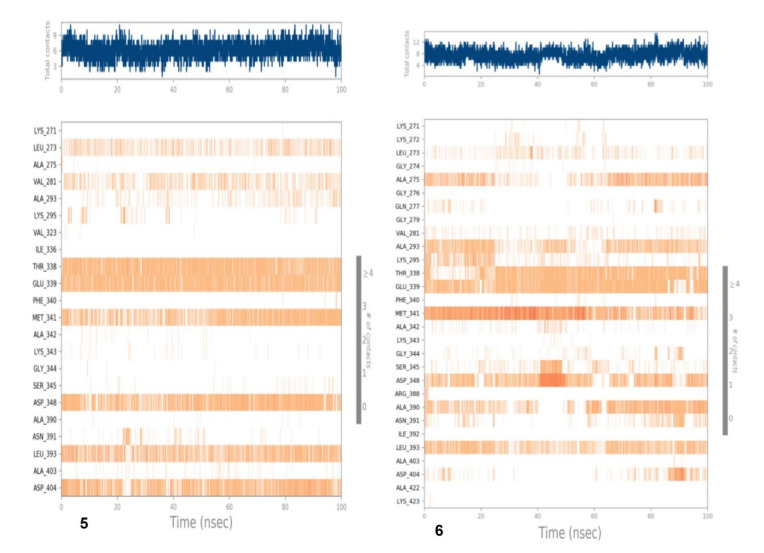
Timeline representation of the interaction and contacts. Top panel shows specific contact of ligand and protein during 100 ns simulation of compounds **5** and **6** with 2HCK.

**Figure 11:**
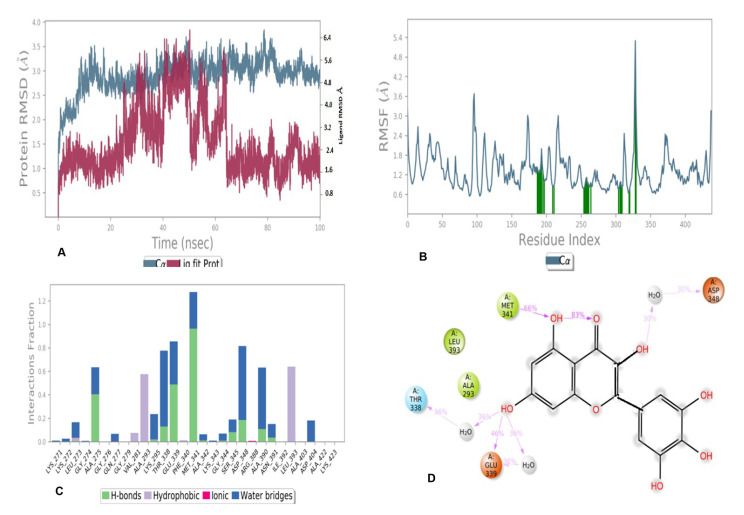
MD simulation results of compound **6** with receptor 2HCK: (**A**) Time-dependent protein-ligand root mean square deviation (RMSD) plots, (**B**) Root mean square fluctuation (RMSF) plots, (**C**) Simulation interactions with bar diagram indicating the fold of interaction, fraction, and contacts, and (**D**) 2D binding interactions.

The time line representation of interactions and contacts for compounds **5** and **6** have been presented in Figure 10. The metrics of root-mean-square deviation (RMSD) for Cα, backbone, and the ligand have been presented in Figure 11A for compound 6 with 2HCK. Root mean square fluctuations for this case have been presented in Figure 11B. Figure 11C demonstrated the interaction fraction analysis of compound 6 with 2HCK during the 100 ns simulation period. Hydrogen bonding was seen at Thr338, Glu339, Met341, and Asp348 and major hydrophobic contact was observed by Leu393 and Ala293. Finally, the 2D trajectory for compound **6** has been presented in Figure 11D.

## 4. Discussion

Six isolated compounds, i.e., 4-methyl salicylic acid, cinnamic acid, chrysin, gallic acid, apigenin, and myricetin from the propolis methanol fraction showed good biological activities. These compounds were also isolated from propolis by different researchers. Salicylic acid is phenolic in nature and is expressed in plant cells, and it is also present in different fruits and vegetables in variable concentrations. This acid is well known for its attributes against inflammation and microorganisms [41]. Touzani, et al. [42] collected propolis from beehives in Morocco and the Palestine territory to conduct various chemical analyses to determine the antioxidant and antibacterial attributes of this valuable bee product. Stompor-Gorący et al. [43] have proposed that the substance chrysin is a form of a group of polyphenols that exist naturally. This substance is also present in fruits, honey, propolis, and other compounds. Chrysin is thought to be involved in different biological mechanisms as it performs a protective role against oxidative stress, neurodegeneration, cancer, and inflammation. Okińczyc, et al. [44] collected Eurasian samples of 19 different types of propolis, mainly from regions in Russia and Kazakhstan, Kyrgyzstan, Poland, Ukraine, and Slovakia. The propolis from poplar constituted chrysin, rutin, and apigenin. Andrade, et al. [45] analyzed the composition of brown, green, and red species of propolis found in the northern states of Alagoas and Sergipe, Brazil, to estimate the type and concentration of biologically active substances. The concentration of phenolic, flavonoid substances was measured, and their antioxidant activity (DPPH) was also examined. Findings showed a high concentration of total phenolics and flavonoid compounds. Red propolis exhibited the highest antioxidant capacity among the three species. For the very first time, bioactive compounds such as artepellin C, acacetin, eriodictyol, gallic acid, isorhamnetin, protocatechuic acid, vanillin, vanillic acid, and isorhamnetin have been reported in Brazilian-based red propolis. Finally, it was proposed that phenolic compounds are mainly responsible for the antioxidant behavior of propolis. Arabian honey bees move from flower to flower in order to collect resin that is ultimately converted into propolis. The presence of these valuable substance in Met depicts Saudi-based propolis’ valuable attribute in the studied parameters.

### 4.1. Total Phenolic Contents (TPC)

It is well-established in various pieces of literature that the reagent Folin-Ciolcateau is not only bound to react with phenols, but it may exhibit its reactive nature with various other reducing substances and may induce their oxidation. Phenols were characterized as a chief origin of compounds that exhibit pharmacological attributes [46]. Propolis samples taken from Met manifested the highest estimates of total phenols when compared to other countries’ propolis samples. A greater quantity of total phenols was also established in various other studies. Different research was organized on phenolic compounds’ phenolic composition in samples based upon propolis [4].

### 4.2. Total Flavonoid Contents (TFC)

TFC results of Saudi-origin propolis were in accordance with propolis from China, India, Macedonia, and Iran where it was found in the range of 08 to 188 mg/g in previous studies by Anandjiwala, Bagul, Parabia, and Rajani [25] and Yang et al. [47]. The concentration of TFC was low in this study as compared to Kumazawa, Taniguchi, Suzuki, Shimura, Kwon, and Nakayama [22], who analyzed American-, Brazilian, Thai-, and New Zealand-based propolis and noted the highest value of 200 mg QE/g of propolis extract. Our findings of TFC are in contrast with the results of [46,48], who conducted a chemical analysis (profiling) and studied the biological actions of propolis obtained from different regions of Guanajuato, Mexico. They found TFC in the range of 13–347 mg QE/g of PE (propolis extract). Flavonoids contents present in extracts of propolis can be credited to local flora from where honey bees take propolis and the climate of a region [46,48].

### 4.3. Free Radical Scavenging Activity

Oxygen is part of numerous vital metabolisms of living things which, as a result, tend to release reactive species of oxygen (ROS). These species of oxygen vehemently take part in the signaling processes of cells and the homeostasis process. However, when any kind of environmental fluctuation is encountered, such as exposure to heat or UV radiations, these byproducts of oxygens are produced in excess. That, in turn, may damage vital cell structures. All of it encompasses oxidative stress [49]. Free radicals are responsible for the uncontrolled oxidation process, and also possess the capability to oxidize all higher-level biomolecules. These reactive species of oxygen, once produced, diffuse away from their original location to other cells, thus enhancing the damaging of many cells [50]. Many edible natural food substances such as veggies, fruits, spices, herbs, and some beehive products (honey, propolis, royal jelly) possess various types of antioxidants that can mitigate the harmful damages caused by oxidative stress to cells. Hex, BuT, Eac, and Met were analyzed for their scavenging capability of DPPH radicals. This radical is purple and belongs to the class of stable organic nitrogen radicals (SONR). The assessment was done on the basis of computing the decrease in the DPPH radicals after reacting with propolis extracts under consideration. It is recognized as the earlier process involved in electron transfer. The property to scavenge above mentioned radical by our tested samples is computed by a parameter known as IC50. It is estimated as the quantity of an antioxidant required to minimize the prior DPPH radicals concentration by fifty percent [51]. Our findings regarding phenolic and flavonoids are responsible for the antioxidant nature of propolis, whether it is taken from Apis bees or from non-Apis bees and are in concurrence with results of other researchers who have also concluded the same [52,53,54,55,56,57]. Phenolics are credited as influential antioxidants that act as chain-breakers, whereas the scavenging ability of polyphenolics is attributed to the presence of a hydroxyl group in their structural composition. Phenolics constituting propolis extract may involve directly to the antioxidant process.

### 4.4. Antimicrobial Activity

Various plant species exhibit antimicrobial nature due to the synthesis of certain substances during secondary metabolism. These substances encompass phenolics and tannin, which are useful against microorganisms and also carry out different biological processes [58]. Bees wander from plant to plant and even at different parts (leaves, buds, and exudes) of the same plant to collect resins for propolis synthesis there; it comprises the majority of the plant’s secondary metabolites. High AMA for Eac and Met may be due to the presence of more number and variety of active chemicals as compared to other extracts. Gram (+) bacterial strains were more prone to propolis extracts than gram (−) strains. These findings are in correlation with the interpretations of Ghanem [30] and Torres, Sandjo, Friedemann, Tomazzoli, Maraschin, Mello, and Santos [33]. Many scientists have tried to explain the mechanism of propolis action against microbes. Fernandes Júnior et al. [59] concluded that the synthesis of proteins is retarded by the active ingredients present in propolis. Moreover, these active compounds also affect the process of division in microbes. Torres, Sandjo, Friedemann, Tomazzoli, Maraschin, Mello, and Santos [33] showed that the cell membrane of the microbes is disrupted by the propolis, causing the release of various intracellular molecules out of the cell, a process known as cell lyses.

### 4.5. Molecular Docking

The binding of compounds **1**–**6** with 2HCK showed that Met341, Thr338, Ala390, Leu273, and Glu339 residues played an active role in hydrogen bonding, whereas the other residues were found to show various type of interactions (Figure 5). The lowest binding energy was observed for Compound 6 as compared to other ligand-receptor complexes. The native ligand showed a binding energy score of −9.3 Kcal/mol, reflecting that the binding affinity of compounds 3, 5, and 6 was almost comparable to the native ligand. The docking results for ligands with 5D6P deciphered that the ligand showed efficient in-silico interlinkages comprising a similar area of the binding pocket beneath hydrophobic and H-bond interplay. The major interacting residues of active site comprised of Ser55, Glu58, Asn54, Ile86, Pro87, Ile51, Arg84, Ile175, Gly85, and Asp81 have a significant role in the binding of ligands. The residues of 5D6P engrossed in H-bonding and associated with compounds **1**–**6** include Arg 84, Asp 81, Ile 51, Glu 58, and Gly 85, whereas Ile 86 and Pro 87 were involved in hydrophobic interactions. The results revealed that compounds **3**, **5,** and **6** possess minimum binding energy with effective interlinkages. Compound **3** in Figure 6 depicts 2 H-bonds to Gly85. Compound 1 showed less affinity towards the active site of 5D6P, whereas compound 5 showed a binding affinity score of −7.6 and delineated conventional linkage with Gly85 and Ile 51 residues with a BD (bond distance) of 2.31Å and 3.07Å. Various other residues taking part in complex interplay were Ile175, Pro87, Ile86, and Glu58. Compound 6 made 5 H-bonds with Glu58, Gly85, Arg84, Ile51, and Asp81 with a bond distance of 2.61, 2.28, 2.84 Å, 3.08, and 2.69 Å, respectively. Various residues which take part in interplay are Ile86 and Pro87.

**Figure 12:**
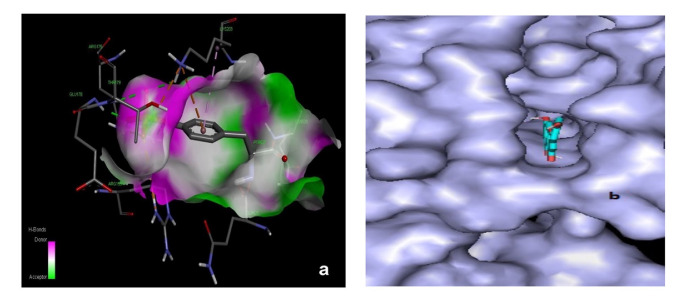
(**a**,**b**): (**a**) Demonstrating Compound **6** in cavity of 5d6p (**b**) Compound **6** in the cavity of 2HCK.

Compound 4 depicted 2 H-bonds with two residues (Gly85, Asp81) with a distance of 2.32 and 3.39Å., respectively. Ile86 and Ile 175 displayed Pi-alkyl associations. Deductions from our findings depicted that compounds **1**–**6** found in Saudi-based-propolis may prove lethal against bacteria and can be deployed to upgrade the antibacterial nature of substances. Compound 6 entering the cavity of 5D6P has also been demonstrated in Figure 12a whereas Figure 12b depicts the pose of compound 6 into the cavity of 2CHK. Interpretation of our docking findings exhibited the valuable antioxidant capability of the compounds isolated from Saudi propolis.

#### 4.5.1. Molecular Dynamic Simulation of Compound **5** and **6** with 5D6P

**Figure 13:**
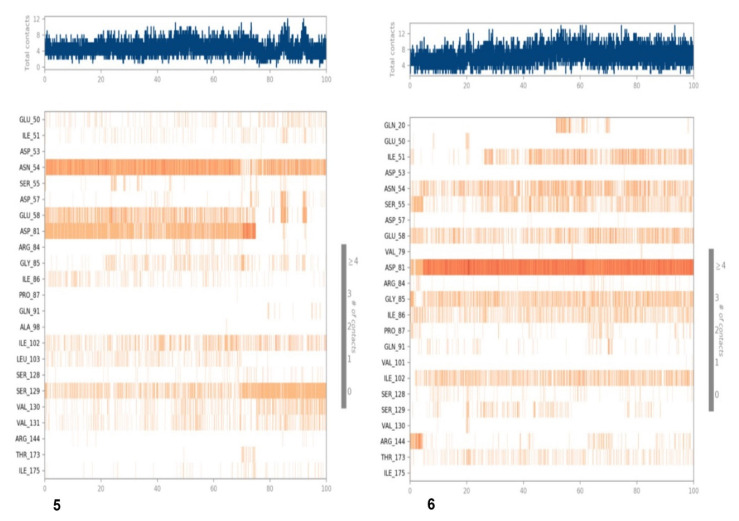
Timeline representation of the interaction and contacts.Top panel shows specific contact of ligand and protein during 100 ns simulation of compounds **5** and **6** with 2HCK.

The RMSD number for the lig fit prot of compound **5** with respect to its initial position increased to 0.9A and remained stable for 70 ns of time scale and increased at a 75, 90, and 95 ns simulation trajectory. The Cα atoms of the backbone fluctuated in the range of 1.25–2.4 Å and higher fluctuations up to 2.4 Å in RMSD were observed at 45–50 ns and 90–95 ns. The flexibility of residues on ligand binding was analyzed using metrics of root mean square fluctuations (RMSFs) in Figure 7B. The RMSF of most of the amino acid residues were within 2.0 Å and only Val70 showed a slightly higher fluctuation at 4.5 Å. High fluctuations were observed in the N- and C terminal region compared to any other part of the protein. The 2D trajectory diagram depicts that the oxygen atom of the carbonyl group at C-4 of chromene accepted one hydrogen bond from Asn54 with 82%, whereas the hydroxyl group at C-5 donated a hydrogen bond to Asp81 with 60%, the hydroxyl group at C-7 of the chromene ring donated a hydrogen bond to Glu58 with 33% of simulation time, and the hydroxyl group at the phenyl ring accepted one hydrogen bond from Ser129 with 41% of simulation time, as shown in Figure 7D. Protein ligand contact showed that Asn54 formed a hydrogen bond throughout the simulation time whereas Asp81 retained a hydrogen bond up to 75 ns of simulation time as shown in Figure 13.

The curve for compound **6** exhibited a fluctuation of 4.8 Å in the case of Lig fit Prot, whereas the backbone showed minor fluctuations up to 60ns of time, a higher fluctuation of 60–70 ns and become stable after 70ns of simulation time in the range of 0.1 to 2.8 Å. The RMSF of most of the amino acid residues were within 1.5 Å and only Val 70 showed a slightly higher fluctuation at 2.3 Å (Figure 8B). High fluctuations were observed in the N- and C terminal region compared to any other part of protein.

#### 4.5.2. MD Simulation of Compound **5** and **6** with 2HCK

It was observed that protein ligand binding for compound **5** was overall stable during the whole simulation time. Some fluctuation in protein was observed during 30–40 and 75–80 ns of time within the range of 4.2 Å. In the case of the ligand, fluctuation was also observed, and that might be due to hydrogen bond formation and it was further reflected from Lig-fit-prot that the RMSD of **5** was lower than the protein throughout simulation time; thus, it can be concluded that compound **5** never moved away from the initial binding site and the interaction was stable, as shown in Figure 9A.

As far as RMSF is concerned, high fluctuation was observed by Asp348, as seen inFigure 9B. The interaction fraction analysis of compound 5 with 2HCK during the 100 ns simulation period has been shown in Figure 9C. This was further evaluated by Figure 9D where the actual percentage of interaction was established between protein and ligand during simulation. The hydroxyl group at C-4 of the chromen ring accepted one hydrogen bond from Thr338 with 88% simulation time and donated a hydrogen bond to Glu339 during 89% of the simulation time, whereas the hydroxyl group at C-5 donated a hydrogen bond to Asp404 through water contact with 46% simulation time whereas another bond was observed between carbonyl oxygen and Met341. The hydroxyl group at the phenyl ring acted as a hydrogen bond donor to Asp348 with 64% of the total simulation time. Figure 11 presented various types of contacts such as H bond, hydrophobic, ionic, and water bridges between proteins and ligands during simulation. The top panel indicated the sum of specific interaction protein makes with the ligand over the course of the trajectory. Specific residues’ interaction with the ligand in each trajectory frame was shown in the bottom panel. Sometimes more than one specific contact with ligands were formed with some residues, which was represented by the dark orange shade. In this figure, it is clear that Thr338 and Glu339 remained in a hydrogen bond interaction throughout the simulation time. Minor fluctuations in backbone protein were observed during the entire simulation time within the range of 2.5-to3.5 Å. The ligand remained stable till 20 ns of simulation time; thereafter, a high fluctuation was observed at 35–65 ns. After 65 ns of simulation time, the ligand became stable [Figure 11A]. As far as RMSF is concerned, root mean square fluctuations demonstrated the critical residues involved in interactions. The essential residues that have hydrogen bonds with docked compound 6 were displayed by the green bar during 100 ns simulation time as shown in Figure 11B. Major fluctuation was observed by Asp348 with RMSF of 4.8 Å. This was further evaluated in Figure 11D where the actual percentage was presented between the docked compound and the protein. Glu339 was seen to interact 46% of the time and Met341 interacted for 66% of the simulation time through the hydrogen bond. Other residues which formed the hydrogen bond through water contact are Thr338 and Asp348. Various types of contacts such as H bond, hydrophobic, ionic, and water bridges between the protein and ligand during simulation have been observed (Figure 13). In this figure, it is clear that Glu339 and Met341 remained in hydrogen bond interaction throughout the simulation time.

## 5. Conclusions

In the current study, a total of six compounds **1**–**6** were isolated from Saudi propolis-based fractions of methanol. The Folin-Ciocalteu colorimetric procedure was deployed to estimate TPC (total phenolic content) and TFC (total flavonoid content) within sub-portions (Hex, Eac, BuT, and Met) that were soluble. The present investigation confirmed after extensive molecular docking the presence of various substances (apigenin, chrysin, gallic acid, 4-methyl salicylic acid, myricetin, cinnamic acid) that are antimicrobial and antioxidant. The Asir region of Saudi Arabia is enriched with various types of propolis that exhibit variations in their chemical constitution that are still to be explored. The components of Saudi propolis accountable for its lethality against microbes and oxidants have been identified by employing the molecular docking methods of separated compounds against 5d6P & 2CHK. The promising stability of receptor-ligand binding has been authenticated through MD simulation studies. Therefore, the findings have clearly established compounds **3**,**5**, and **6** as promising compounds against the harmful effects of oxidants and microbes.

## Figures and Tables

**Table 1:** TFC (flavonoid contents), TP (total phenolics), DPPH (2,2-diphenyl-1-picryl-hydrazyl) IC_50_, and Ferric Reducing Antioxidant Power (FRAP) of various Arabian propolis extracts.

Extract	Total Polyphenol (mgGAE/g Extract)	Total Flavonoids (mg QE/g Extract)	DPPH (IC_50_) ug/mL	FRAP (mg Fe(II)/g
Hex	225.33 ± 4.04 d	34.04 ± 1.45 d	249.01 ± 2.09 a	8.75 ± 0.62 a
Eac	305.67 ± 4.16 b	93.22 ± 1.32 b	130.51 ± 3.16 c	5.32± 0.51 d
BuT	271.67 ± 3.21 c	84.82 ± 2.15 c	176.31 ± 3.36 b	6.31± 0.23 b
Met	325.00 ± 3.61 a	96.65 ± 0.87 a	111.36 ± 2.41 d	5.81± 0.48 c
Ascorbic Acid (Positive control)	-	-	17.72 ± 3.17 e	

Every value is mean ± SD, standard deviation. GAE (Gallic acid equivalent), QE (Quercetin equivalent). Hex, hexane extract; Eac, ethyl acetate extract; BuT, butanol extract; and Met, methanol extract of Saudi Propolis. IC_50_ = Half maximal inhibitory concentration. Means indicated with different letters in the same column are statistically different (*p* ≤ 0.05).

**Table 2:** Consequences of quantity (µg/mL) on antioxidant behavior of different Saudi propolis-based extract.

Propolis Extracts	Antioxidant Activity (% age)									
	Concentration µg/mL									
	5	10	20	40	60	80	100	150	200	250
Hex	1.87 ± 1.08 b	3.22 ± 2.12 d	9.71 ± 2.59 c	13.48 ± 3.54 c	16.57 ± 2.24 e	20.26 ± 3.35 e	28.51 ± 1.29 e	35.89 ± 1.47 e	39.66 ± 0.55 e	40.06 ± 3.04 e
Eac	5.7 ± 2.46 b	13.34 ± 2.66 b	20.20 ± 2.74 b	27.60 ± 2.06 b	34.46 ± 0.97 c	41.96 ± 1.33 c	50.45 ± 1.07 c	61.69 ± 2.12 c	68.23 ± 3.25 c	72.47 ± 1.01 c
BuT	1.55 ± 1.40 b	4.17 ± 1.00cd	10.56 ± 0.98 c	17.58 ± 1.16 c	21.78 ± 0.54 d	31.55 ± 1.45 d	39.44 ± 0.86 d	48.48 ± 0.70 d	55.27 ± 0.55 d	60.29 ± 1.82 d
Met	6.40 ± 1.13 b	10.60 ± 1.20bc	23.08 ± 2.01 b	30.69 ± 0.76 b	38.83 ± 1.81 b	51.51 ± 2.76 b	59.03 ± 3.39 b	67.41 ± 1.86 b	75.04 ± 1.11 b	79.13 ± 1.30 b
AA	33.15 ± 2.58 a	42.34 ± 1.80 a	52.67 ± 1.45 a	58.62 ± 2.79 a	64.87 ± 1.66 a	71.11 ± 2.17 a	77.98 ± 0.65 a	87.63 ± 2.54 a	91.80 ± 1.63 a	98.15 ± 0.60 a

Each value is mean ± standard deviation. Hex, hexane extract; Eac, ethyl acetate extract; BuT, butanol extrac; AA, Ascorbic Acid; and Met, methanol extract of Saudi propolis. a–e Means indicated with a different letter in the same column are statistically different (*p* ≤ 0.05).

**Table 3:** Minimum inhibitory concentration (MIC) of the various propolis extracts against microbial strains.

Microorganisms	MIC (mg/mL)				
Gram-Negative Bacteria	Hex *	Eac *	BuT *	Met *	CTL *
*Escherichia coli* ATCC 25922	2.50	1.25	2.50	2.50	0.312
*Shigella flexneri* ATCC 12022	2.50	2.50	1.25	1.25	1.25
*Proteus mirabilis* ATCC 29906	1.25	0.625	1.25	2.50	0.625
**Gram-positive Bacteria**					
*Staphylococcus aureus* ATCC 29213	1.25	1.25	2.50	0.625	2.50
*S. epidermidis* ATCC 12228	2.50	0.625	1.25	0.625	0.312
**Fungus**					
*Candia albicans* ATCC 7596	2.50	1.25	1.25	2.50	1.25

* Hex, Eac, BuT, Met = Propolis extracts with hexane, ethyl acetate, butanol, and methanol solvents, respectively. CTL = Dimethyl sulfoxide (DMSO) as control.

## Data Availability

Data is contained within the article and Supplementary Materials.

## Supplementary Materials

The following supporting information can be downloaded at: https://www.mdpi.com/article/10.3390/antiox11071413/s1, Table S1: ^1^H-NMR values of compounds **1**–**6** separated from Met (Methanolic fraction).
