# Supplementary material for: Chemical Profiling, Antioxidant, and Antimicrobial Activity of Saudi Propolis Collected by Arabian Honey Bee (Apis mellifera jemenitica) Colonies
Source: Antioxidants (Basel). 2022 Jul 21;11(7):1413. doi: 10.3390/antiox11071413 (PMC9311549; doi:10.3390/antiox11071413)
Supplement: Supplementary file 1 [file antioxidants-11-01413-s001.zip › antioxidants-1818464-supplementary.pdf]

antioxidants MDPI

Table S1. ^{1}H-NMR values of compounds **1-6** separated from Met (Methanolic fraction).

4-methyl salicylic acid (1)	Cinnamic acid (2)	Chrysin (3)	Gallic Acid (4)	Apigenin (5)	Myricetin (6)
6.72 (1H, s, H-3)	6.72 (1H, m, H-2)	6.41 (1H, s, H-3)	6.91 (1H, s, H-2)	6.72 (1H, s, H-3)	-
-	6.12 (1H, m, H-3)	-	-	-	-
6.12 (1H, d, H-5)	6.12 (1H, m, H-4)	-	-	-	-
6.41 (1H, d, H-6)	6.41 (1H, m, H-5)	6.12 (1H, d, J = 2.2 Hz, H-6)	6.91 (1H, s, H-6)	6.41 (1H, d, J = 2.1 Hz, H-6)	6.15 (1H, d, J = 2.2 Hz, H-6)
2.34 (s, 3H, -CH3)	6.12 (1H, m, H-6)	6.51 (1H, d, J =2.2 Hz, H-8)	-	6.12 (1H, d, J = 2.1 Hz, H-8)	6.39 (1H, d, J = 2.2 Hz, H-8)
-	5.89 (1H, d, J = 14.5 Hz, H-2′)	8.1 (1H, d, J= 8.4, H-2′)	-	7.89 (1H, d, J = 8.5 Hz, H-2′, H-6′)	6.91 (2H, s, H-2′, H-6′)

-	5.90 (1H, d, J = 14.5 Hz, H-3′)	7.49 (1H, m, H-3′)	-	6.90 (1H, d, J = 8.5 Hz, H-3′, H-5′)	-
-	-	7.49 (1H, m, H-4′)	-	-	-
-	-	7.49 (1H, m, H-5′)	-	-	-
-	-	8.1 (1H, d, J= 8.4, H-6′)	-	-	-
9.89	9.29, 6.28	10.55, 12.36	10.55, 12.36	10.29, 12.28	10.99, 11.33
